# The State Legislature and Vivisection

**Published:** 1875-01

**Authors:** 


### The State Legislature and Vivisection.

Physicians and students of medicine have reason to believe that an attempt will be made by certain overzealous members of the Society for the Prevention of Cruelty to Animals, to secure a repeal of the clause in the present law for the prevention of cruelty to animals, which declares that "*Nothing in this act shall be construed to prohibit or interfere with any properly coducted, scientific experiments or investigations.*"

The persons who are attempting to secure the repeal or modification of this law, are doubtless actuated by the best motives, but are really ignorant of what they are seeking to accomplish. If the law, which they are seeking to establish, had been in operation for the past fifty years, most of the important and valuable discoveries, of which medical men are so proud, would have remained unknown, and we should be still practicing medicine according to the unreasoning routine of the middle ages.

The persons who are most active in securing the establishment of legal restrictions of vivisection, are the very ones who are most ignorant of the necessity of such experimental investigations to the progress of medical science. We are positive that there cannot be found an educated physician who will sanction their effort; and no person of scientific education will, upon giving the subject a moment's thought, lend his support to a movement which is calculated so seriously to interfere with medical study.

We are earnestly in favor of the prevention of wanton cruelty to animals, and we aver that the physiologist does not live who would inflict pain upon any of the lower animals from sheer cruel intention—his knowledge of the intricate mechanism of animal life would rather lead him to prevent the infliction of such pain. He who sees a living being suffering from pain, and does not make some effort to relieve it, is as guilty of cruelty as the one who from wantonness inflicts pain and suffering. The alleviation of disease and pain, is the great problem which physiologists are seeking to solve, and the man, or body of men, who places obstacles in their path, is guilty of a crime against the human race.

We are pleased to notice that various county hnd district medical societies have taken this matter in hand, but the passage of resolutions by societies is of little benefit, unless the individual members of those societies will urge upon the representatives from their districts the necessity of continning this clause in force.

